# [Retracted] NF-κB interaction long non-coding RNA inhibits migration, invasion and epithelial-mesenchymal transition of cervical cancer cells through inhibiting NF-κB signaling pathways 

**DOI:** 10.3892/etm.2025.12984

**Published:** 2025-09-29

**Authors:** Feipeng Wang, Xiaochun Jiang, Pei Wang

Exp Ther Med 20:1039–1047, 2020; DOI: 10.3892/etm.2020.8752

Following the publication of this paper, it was drawn to the Editors’ attention by a concerned reader that certain of the western blotting data shown in Fig. 5A on p. 1045 were strikingly similar to data that had already appeared in different form in another article written by different authors at different research institutes, which was published in the journal *OncoTarget*, although that paper has subsequently been retracted.

In view of the fact that the abovementioned data had already apparently been published previously, the Editor of *Experimental and Therapeutic Medicine* has decided that this paper should be retracted from the Journal. The authors were asked for an explanation to account for these concerns, but the Editorial Office did not receive a reply. The Editor apologizes to the readership for any inconvenience caused.

